# Systemic treatment of hormone receptor positive, human epidermal growth factor 2 negative metastatic breast cancer: retrospective analysis from Leeds Cancer Centre

**DOI:** 10.1186/s12885-020-6527-y

**Published:** 2020-01-21

**Authors:** Chris Twelves, Sue Cheeseman, Will Sopwith, Matthew Thompson, Majid Riaz, Necibe Ahat-Donker, Melissa Myland, Adam Lee, Raymond Przybysz, Stuart Turner, Geoff Hall, Tim Perren

**Affiliations:** 1 Clinical Cancer Pharmacology and Oncology, Leeds Cancer Centre, St James’s University Hospital, Level 4, Bexley Wing, Beckett Street, Leeds, LS9 7TF UK; 2 University of Leeds, Leeds, UK; 3 IQVIA, London, UK; 4 Novartis Pharmaceuticals UK Ltd, Surrey, UK; 5 Novartis Pharmaceuticals Corporation, East Hanover, NJ USA

**Keywords:** Metastatic breast cancer, HR+/HER2-, Chemotherapy, Endocrine therapy, Overall survival

## Abstract

**Background:**

Study aimed to characterise treatment and outcomes for patients with hormone receptor positive (HR+), human epidermal growth factor 2 negative (HER2-) metastatic breast cancer (MBC) within a large regional cancer centre, as a benchmark for evaluating real-world impact of novel therapies.

**Methods:**

Retrospective longitudinal cohort, using electronic patient records of adult females with a first diagnosis of HR+/HER2- MBC January 2012–March 2018.

**Results:**

One hundred ninety-six women were identified with HR+/HER2- MBC. Median age was 67 years, 85.2% were post-menopausal and median time between primary diagnosis and metastasis was 5.4 years. Most (75.1%) patients received endocrine therapy as first line systemic treatment (1st LoT); use of 1st LoT chemotherapy halved between 2012 and 2017. Patients receiving 1st LoT chemotherapy were younger and more likely to have visceral metastasis (*p* < 0.01). Median OS was 29.5 months and significantly greater for patients with exclusively non-visceral metastasis (*p* < 0.01). The adjusted hazard ratio for death of patients with visceral (or CNS) metastasis was 1.91 relative to those with exclusively non-visceral metastasis.

**Conclusions:**

Diverse endocrine therapies predominate as 1st LoT for patients with HR+/HER2- MBC, chemotherapy being associated with more aggressive disease in younger patients, emphasising the importance of using effective and tolerable therapies early.

## Background

Breast cancer (BC) is biologically heterogeneous, and genomic signatures are increasingly recognised [1]. Clinical decisions regarding systemic anticancer treatment (SACT), however, are usually influenced by expression of oestrogen/progesterone hormone receptor (HR) and human epidermal growth factor receptor 2 (HER2) status [2] along with patient preference, prior therapy (and tolerability), comorbidities, and organ function [3].

An estimated 60–75% of patients with MBC have HR+/HER2- disease [4]. Where rapidly progressing visceral disease is absent, the UK National Institute for Health and Care Excellence recommends endocrine therapy as first-line systemic treatment (1st LoT) for such patients [3], mirroring international recommendations [5–7].

Until recently, 1st LoT endocrine options comprised selective estrogen receptor modulators (e.g. tamoxifen), selective estrogen receptor downregulators (e.g. fulvestrant) or (in post-menopausal women) third generation aromatase inhibitors (AI; e.g. anastrazole, letrozole or exemestane) [3]. Subsequently, guidelines have recommended further endocrine therapy, usually with a different class of agent, unless rapid disease progression in a patient raises concerns regarding endocrine resistance, or a patient has rapidly progressive visceral disease. The NCCN recommend continuing endocrine therapy for a maximum of 3 regimens until progression or unacceptable toxicity [7].

The treatment landscape for HR+/HER2- MBC has, however, changed substantially in recent years with the advent of targeted therapies combined with endocrine therapy. Administration of the mTOR inhibitor everolimus in combination with exemestane results in a significant gain in progression free survival (PFS); improvement in overall survival (OS) did not, however, achieve statistical significance and the combination is associated with significant additional toxicity [8]. A more recent target is cyclin-dependent kinase 4/6 (CDK 4/6); a number of highly selective CDK 4/6 inhibitors have been licensed and are becoming widely available [9]. Trials of palbociclib, ribociclib and abemaciclib, in combination with various endocrine therapies, have shown a consistent and statistically significant doubling of PFS in the first line metastatic setting, with a lesser, but clinically significant, prolongation of OS [10, 11]. Similar benefits are seen in the second line metastatic setting [12–14]. Importantly, tolerability of CDK 4/6 inhibitors is generally good, the principle toxicities being asymptomatic neutropenia (palbociclib and ribociclib) or diarrhea (abameciclib). The combination of a CDK 4/6 inhibitor with first or subsequent lines of endocrine therapy, or the use of everolimus in combination with exemestane following progression on a non-steroidal AI, are now considered standards of care [15–17].

Gaining a fuller understanding of current clinical practice and patient outcomes is important in understanding the likely impact of these new therapies. There is, however, relatively little published information on real-world treatment patterns of patients with HR+/HER2- MBC prior to the introduction of CDK 4/6 inhibitors. The aim of this study was to characterise these patients and describe outcomes within a large regional cancer centre, as a benchmark against which the real-world impact of novel therapies can be evaluated. Study objectives include scoping the potential for more effective treatment with an AI combined with a CDK 4/6 inhibitor to replace chemotherapy as 1st LoT SACT for patients with HR+/HER2- MBC, or to delay its use until later in the disease course.

## Methods

### Study design

This study was conducted using a retrospective longitudinal cohort design, with secondary use of de-identified, coded and uncoded hospital electronic patient records (EPR). Patients with a first diagnosis of HR+/HER2- MBC made between January 2012 and March 2018 were identified from a major regional NHS cancer centre. There was no pre-defined minimum period of follow-up, which varied according to the date of entry into the study cohort.

### Setting

The Leeds Cancer Centre (LCC) serves a metropolitan catchment area of over 850,000 people for secondary care at Leeds Teaching Hospitals NHS Trust (LTHT). Patient pathway manager (PPM) is an in-house LTHT hospital EPR in routine clinical use since 2003.

This study was conducted by Real-world Evidence Alliance Leeds (REAL) Oncology, a collaboration between LCC, Leeds Institute for Data Analytics (University of Leeds) and IQVIA Real World Insights. REAL Oncology accesses continually updated patient data stored in PPM, including patient demographics, cancer diagnoses, tumour staging and anti-cancer therapy. REAL Oncology studies are conducted on-site within LTHT under the strict legal framework governing access to and use of personal information in the NHS [18].

### Patients and methods

Eligible patients were adult females (≥ 18 years old) with a first diagnosis of HR+/HER2- MBC made during the study period and whose treatment for MBC was overseen or administered at LTHT. They comprised patients with a first relapse after a previous diagnosis of early stage BC, defined as ‘recurrent MBC’, and those with metastatic disease at their initial diagnosis of BC, defined as ‘de novo MBC’.

A two-step process of participant identification was used. A potential cohort was identified from coded fields in PPM, defining MBC by the International Classification of Disease (ICD)-10 code C50, and American Joint Committee on Cancer (AJCC) stage IV. Where HR or HER2 receptor status was missing in coded fields, past treatment with endocrine therapy was used as a proxy for HR+ status and these patients were included; treatment with trastuzumab was used as a proxy for HER2+ status and these patients were excluded. Uncoded EPR for this potential cohort were then manually reviewed by a consultant oncologist to update missing variables in coded fields including confirmation of HR+/HER2- status.

Patients treated within a clinical trial (prior to or during study period) or receiving CDK 4/6 inhibitors (before their reimbursement) were excluded.

### Data variables

Index date was that of first diagnosis with MBC. Follow-up was defined as the interval between index date and the confirmed date of death, the censor date (if lost to follow-up) or study end in March 2018. Where patients progressed to MBC following a previous diagnosis of early stage disease, the metastasis-free interval (MFI) was defined as the time in months between primary diagnosis of BC and the first diagnosis of metastatic disease.

Unless otherwise stated, patient and clinical characteristics were as recorded in PPM at index date, or the date closest to (and following) index date. TNM staging was according to the Union for Cancer Control (UICC)/AJCC TNM classification (7th edition). Histology was defined in accordance with ICD-10- morphology codes and grade. Where menopausal status was not recorded in coded fields or in the uncoded EPR, patients aged 55 years and over were classified as post-menopausal and those aged less than 55 were classified as of unknown menopausal status.

Where receptor status was missing in coded fields, HR+/HER2- receptor status was derived from uncoded EPR and was defined as HR positive if the pathology report stated ‘positive’ or either oestrogen or progesterone receptor status score was at least 3 (out of 8) using immuno-histochemistry (IHC); HER2 was negative if the pathology report stated ‘negative’, receptor status score was 0 or 1 by IHC, or score 2 by IHC but negative by in-situ hybridisation (ISH).

Start dates and planned end dates of chemotherapy administered to patients were available in PPM. Some endocrine treatments were available in PPM but full data on endocrine therapy was collected from uncoded EPR to ensure all prescriptions were included. Where end date of endocrine therapy was missing, and no subsequent treatment was recorded, patients were considered to be still receiving endocrine therapy until end of follow up. Treatment with SACT was reported by regimen, modality (endocrine therapy, endocrine/targeted therapy [i.e. everolimus plus exemestane], or chemotherapy) and LoT. First LoT was designated as first SACT given with palliative intent following index date. Sequential LoT treatment was defined as a different therapy received after the start date of previous treatment. Endocrine therapy potentially prescribed as “maintenance” following completion of a chemotherapy regimen was counted as a subsequent LoT, since dates of progression were not extracted. Treatment duration was derived from treatment start and end dates.

Following clinical review, patients were excluded if any one or more of the following applied: undefinable age or sex; incomplete staging information; undefinable HR or HER2 receptor status; incomplete sequence of treatment records (e.g. where an incident diagnosis was made outside the LCC); significant other malignancies present at index date.

### Data analysis

Summary statistics were calculated for categorical and continuous baseline demographic variables as appropriate, including by sub-cohort of metastatic status (‘de novo’ or ‘recurrent’ MBC). Continuous variables were described by the mean, standard deviation, median, and range. Categorical variables were described by the number and percentage of patients in each category. Univariate analyses were performed to explore relationships between variables; differences between categorical variables were tested using Pearson *χ*^2^ test, and differences between continuous variables tested using parametric two sample t-tests, where appropriate. Where trend over time was measured, goodness of fit was reported using R^2^. Significance level was *p* < 0.05 using 2-sided testing. Where sub-cohorts included 5 or fewer individuals, actual numbers were masked in line with local data protection policy to prevent potential identification of individuals.

OS from index date was summarised using the non-parametric Kaplan Meier method, with date of death due to any cause as the end-point. Date of death was confirmed by monthly reconciliation of PPM with Office for National Statistics death certifications. Data were stratified by selected baseline characteristics considered risk factors for survival; log-rank tests were used to compare survival between subgroups. Median OS was reported with 95% confidence intervals (95% CI).

Following testing assumptions, a Cox proportional hazards (PH) regression model was constructed to estimate the effect of various prognostic variables on survival, with person-time follow-up as the underlying timescale. Covariates included in the model were restricted to those where the data were at least 65% complete. A step-down modelling strategy initially included all candidate confounders in the base-model, with parameters not significant at the 0.05 level subsequently dropped. Where there was evidence of strong correlation between covariates retained in the model, each variable in the pair was dropped separately from the global model to assess best fit. The most desirable parsimonious model, i.e. the simplest model with the least assumptions and variables but with greatest explanatory power, was that with the lowest Akaike information criterion, with correction for small sample size.

All analyses were done using SAS v9.4 (SAS, Cary, NC, USA).

## Results

### Baseline demographic and clinical characteristics

The study population was drawn from a prevalent population of 4246 female patients alive with a diagnosis of BC between 1st January 2012 and 31st March 2018. Over this 75-month period, 464 patients were identified with a new diagnosis of locally advanced BC or MBC, 318 of whom (68.5%) had HR+/HER2- disease. In all, 196 women with MBC were eligible for the analysis; 47 (38.5%) of those excluded had locally advanced BC, 35 (28.7%) had participated in a clinical trial, 26 (21.3%) had incomplete records and the remainder had another malignancy at index (Additional file 1).

Patient characteristics for the study cohort are summarized (Table 1). Median age was 67 years and the majority of patients were post-menopausal (*n* = 167, 85.2%). Median follow-up time was 34 months (range 0.3–77). Approximately two thirds of patients (*n* = 124, 63.3%) had recurrent MBC with a median time between primary diagnosis and metastasis of 5.4 years (range 0.5–28); the remainder (*n* = 72, 36.7%) had de novo MBC. Metastatic disease at index date was exclusively non-visceral for 73 patients (37.2%) and exclusively visceral (including CNS sites) for 31 (15.8%); the remaining 88 patients (44.9%) had metastases at both visceral and non-visceral sites. Bone was the most commonly recorded site of metastasis (*n* = 133, 67.9%).

**Table 1:** Selected characteristics of patients with metastatic HR+/HER2- BC

Characteristic	Study cohort (N, %)	Treated with 1st LoT SACT (N, %)
All patients	196	185
Age at index date, median (range)	67 years (33–92)	68 years (33–92)
< 55 years	39 (19.9%)	34 (18.4%)
55–74 years	99 (50.5%)	97 (52.4%)
75+ years	58 (29.6%)	54 (29.2%)
Pre/peri-menopausal^a^	25 (12.8%)	20 (10.8%)
Post-menopausal	167 (85.2%)	161 (87.0%)
Morphology (1° tumour)		
Infiltrating duct carcinoma, NOS^b^	117 (59.7%)	109 (58.9%)
Lobular carcinoma, NOS	29 (14.8%)	28 (15.1%)
Carcinoma, NOS	26 (13.3%)	26 (14.1%)
Other	24 (12.2%)	22 (11.9%)
Non-visceral metastasis only^c^	73 (37.2%)	66 (35.7%)
Bone	59 (30.1%)	54 (29.2%)
Lymph nodes	24 (12.2%)	21 (11.4%)
Skin and soft tissue	17 (8.7%)	15 (8.1%)
Non-visceral with visceral metastasis^c^	88 (44.9%)	86 (46.5%)
Bone	74 (37.8%)	73 (39.5%)
Lymph nodes	44 (22.4%)	42 (22.7%)
Skin and soft tissue	10 (5.1%)	10 (5.4%)
Pulmonary	52 (26.5%)	52 (28.1%)
Liver	40 (20.4%)	39 (21.1%)
Pleura	26 (13.3%)	25 (13.5%)
Peritoneum	10 (5.1%)	9 (4.9%)
CNS	6 (3.1%)	6 (3.2%)
Visceral (incl. CNS) metastasis only^c^	31 (15.8%)	29 (15.7%)
Pulmonary	12 (6.1%)	12 (6.5%)
Liver	16 (8.2%)	14 (7.6%)
Pleura	7 (3.6%)	7 (3.8%)
CNS	< 6	< 6
Metastatic status		
Recurrent metastatic	124 (63.3%)	111 (61.3%)
De novo metastatic	72 (36.7%)	70 (38.7%)

^a^There were < 6 patients for whom menopausal status was not defined

^b^*NOS* Not otherwise specified

^c^Patients may have multiple sites of metastases; categories not mutually exclusive; < 6 patients had metastasis with an unknown site

### Treatment for metastatic disease

Almost all patients (*n* = 192, 98.0%) received SACT during the study period; the median number of distinct treatments with SACT following index date was 2 (range 1–9). At some point, almost all patients received endocrine therapy (*n* = 182, 94.8%) whereas 91 (47.4%) received chemotherapy. Seven patients were omitted from analysis by LoT because initiation of treatment pre-dated confirmation of metastatic disease. Subsequent analyses are based on the remaining 185 patients receiving SACT post-diagnosis of MBC, for whom the distributions of site of metastasis, and de novo and recurrent MBC were similar to the overall cohort (Table 1).

Patients with recurrent MBC were more likely to have visceral metastases (*n* = 75, 62.5%), than those with de novo MBC (*n* = 28, 38.9%; *p* < 0.01). Although visceral metastases were more frequent amongst patients under 55 years than in older age groups (75.8% [*n* = 25] and 60.8% [*n* = 90], respectively), this difference was not statistically significant (*p* = 0.11).

#### Endocrine therapy

Endocrine therapy was the modality most frequently used as 1st LoT (*n* = 127, 68.6%), with a further 12 (6.5%) receiving endocrine/targeted therapy. Median treatment duration of 1st LoT endocrine (with/without targeted) therapy was 382 days (range 13–1708). Patients treated with 1st LoT endocrine most commonly received an AI (114 patients, 82.0%); tamoxifen (8.6%) or exemestane/everolimus in combination (8.6%) were used less frequently (Table 2).

**Table 2:** Treatments received by patients at first line of therapy following diagnosis of metastatic disease, showing the percentage of patients in each modality of therapy

Treatment at 1st LoT	N	% of modality
Endocrine (including targeted)	139	100
Letrozole	56	40.3
Anastrozole	35	25.2
Exemestane	23	16.5
Tamoxifen	12	8.6
Everolimus + exemestane	12	8.6
Other	< 6	
Chemotherapy	46	100
Paclitaxel	16	34.8
Epirubicin + cyclophosphamide	14	30.4
Capecitabine	7	15.2
Carboplatin + paclitaxel	6	13
Other	< 6	

#### Chemotherapy

Forty-six patients (24.9%) received 1st LoT chemotherapy and median treatment duration was 129 days (range 24–278). The proportion of patients receiving 1st LoT chemotherapy appeared to decrease between 2012 and 2017 (33.3 and 15.4%, respectively), although this trend was not statistically significant (R^2^ = 0.38, *p* = 0.19).

No single class of chemotherapy predominated as 1st LoT; 26 patients (56.5%) received single agent chemotherapy (paclitaxel, capecitabine or docetaxel); the remaining 20 received combination chemotherapy (Table 2). Half those patients on combination therapy had recurrent MBC and carboplatin with paclitaxel (*n* = 6) was only used for patients with recurrent MBC who had been treated with (neo)adjuvant chemotherapy. Epirubicin and cyclophosphamide (EC; *n* = 14) was only used for patients with visceral metastasis, whether with recurrent MBC following (neo)adjuvant treatment with endocrine or with de novo MBC.

### Factors influencing use of chemotherapy as 1st line SACT

#### Age and site of metastasis

Patients receiving 1st LoT chemotherapy were younger (median age 59, range 33–84; *p* < 0.01), more likely to be pre/peri-menopausal (*n* = 9, 45.0%; *p* = 0.02) and more likely to have visceral metastasis (*n* = 41, 89.1%; *p* < 0.01) than those receiving endocrine or endocrine/targeted treatment (Table 3). Very few patients with exclusively non-visceral metastasis at index date (< 10%) were treated with 1st LoT chemotherapy.

**Table 3:** Association of age group with site of metastasis and 1st LoT treatment modality for patients treated with 1st LoT SACT

		Age group (years)				*χ2*
		< 55 (*n* = 33)	55–74 (*n* = 95)	75+ (*n* = 53)	total	
All (*n* = 181)	Non-visceral only	8 (24.2%)	39 (41.1%)	19 (35.8%)	66 (36.5%)	*p = *0.223
	Visceral	25 (75.8%)	56 (58.9%)	34 (64.2%)	115 (63.5%)	
Non-visceral mets only (*n* = 66)	Chemo	< 6	< 6	< 6	< 6	*p = *0.495^a^
	Endo +/−targ	7 (87.5%)	37 (94.9%)	17 (89.5%)	61 (92.4%)	
Visceral mets (incl. brain) (*n* = 115)	Chemo	17 (68.0%)	22 (39.3%)	< 6	41 (35.7%)	*p *< 0.001
	Endo +/−targ	8 (32.0%)	34 (60.7%)	32 (94.1%)	74 (64.3%)	
All (*n* = 181)	Chemo	18 (54.5%)	24 (25.3%)	< 6	46 (25.4%)	*p *< 0.001
	Endo +/−targ	15 (45.5%)	71 (74.7%)	49 (92.5%)	135 (74.6%)	

^a^Freeman-Halton Fisher exact test statistic

*Chemo* Chemotherapy, *Endo +/−targ* Endocrine (with or without targeted) therapy

Of the 115 patients with visceral metastasis, 17 (68.0%) of those aged under 55 years were treated with chemotherapy compared with fewer than 6.0% of those aged 75 years and over (*p* < 0.01) (Table 3). The 66 patients with exclusively non-visceral metastasis were, however, almost always treated with 1st LoT endocrine (with/without targeted) therapy, regardless of their age (Table 3).

#### Mode of presentation with MBC

Patients with recurrent MBC were somewhat more likely to be treated with 1st LoT chemotherapy (*n* = 31, 27.4%) than those with de novo MBC (*n* = 15, 20.8%); this difference in treatment modality was not, however, statistically significant (*p* = 0.31).

#### Influence of prior (neo)adjuvant treatment and MFI

All 124 patients with recurrent metastatic disease had received (neo)adjuvant endocrine therapy, with or without (neo)adjuvant chemotherapy. Sixteen patients (12.9%) developed metastatic disease within 24 months of primary diagnosis and 29 patients (23.4%) at least 120 months after that diagnosis; the median MFI was 64.9 months (range 5.6–339.2). This implies that around half of those developing recurrent MBC will have done so whilst on (neo)adjuvant endocrine therapy. Those with MFI longer than average will have been progressively less likely to have relapsed whilst receiving (neo)adjuvant endocrine therapy.

The length of MFI was associated with young age at index date, patients aged under 55 years having the shortest MFI (*p =* 0.01); duration of MFI was not, however, associated with the likelihood of developing visceral metastasis (*p* = 0.93). 1st LoT chemotherapy was significantly more common than 1st LoT endocrine therapy in patients with an MFI less than 60 months (61.3 and 40.2%, respectively; *p* = 0.03*),* indicating increased use of chemotherapy in those relapsing whilst on (neo)adjuvant endocrine therapy.

### Second LoT and subsequent SACT

**Fig. 1:**
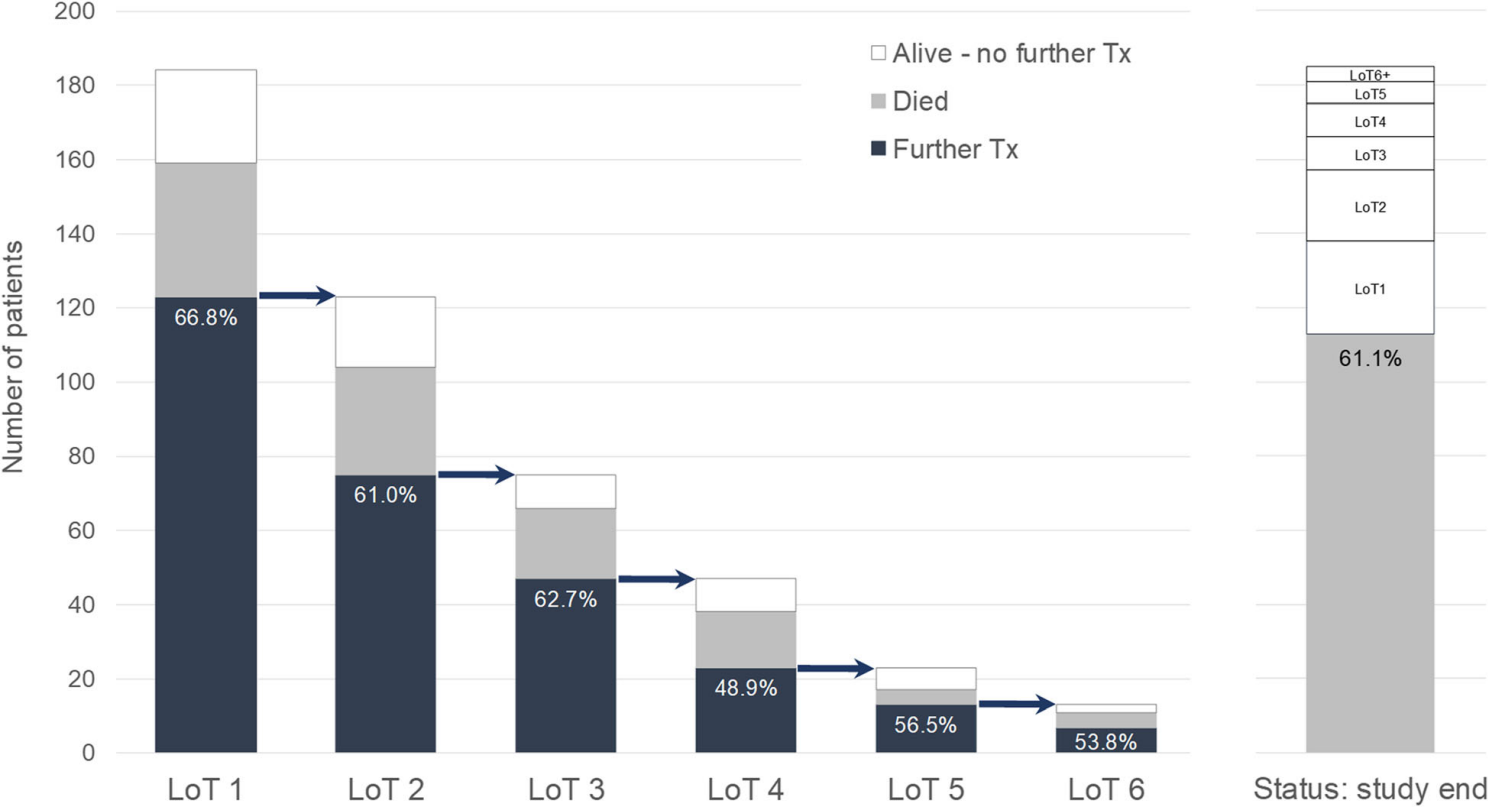
Treatment (Tx) outcomes for each distinct LoT (up to 6 LoT shown), including status at end of study. The percentage of patients receiving subsequent treatment (shown by an arrow between bars) is labelled. The numbers of patients dying before receiving subsequent treatment are distinguished from those remaining alive without a change of treatment before end of study period

Of the 185 patients receiving 1st LoT, 123 (66.5%) received subsequent SACT. The mean proportion changing to further treatment following 1st to 3rd LoT was 64.1%, falling to 51.8% following 4th to 6th LoT (Fig. 1). Of those receiving 1st LoT chemotherapy, 39 (84.8%) had subsequent SACT, compared with 84 (60.4%) of those receiving 1st LoT endocrine (with/without targeted) therapy (*p* < 0.01). The extent to which this subsequent treatment represents “maintenance” endocrine therapy without disease progression is, however, not known.

Following 1st LoT endocrine (with/without targeted) therapy or chemotherapy, 62 patients did not receive subsequent SACT; 36 of these patients (58.0%) died during or following 1st LoT, the remainder either having completed or continuing 1st LoT. Following 2nd LoT SACT, 48 patients did not receive further SACT, 29 (60.4%) having died during or following 2nd LoT. By the end of the study period, 61.1% treated patients had died, with the remainder being alive either on or off SACT (Fig. 1).

**Fig. 2:**
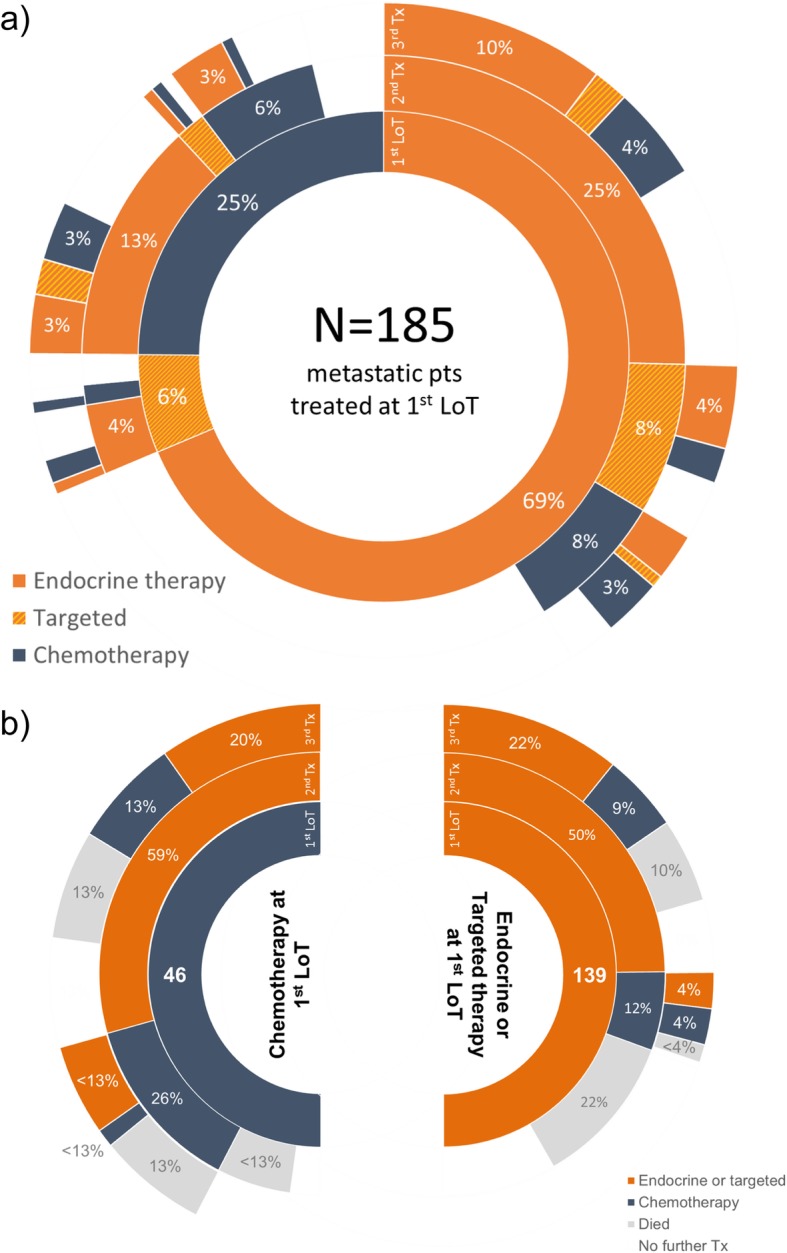
Sequence of treatment (Tx) categories given following diagnosis with metastatic disease, showing treatments from 1st LoT (inner ring) through to a third treatment (outer ring): **a** all treatment category sequence, **b** treatment sequence as proportion of 1st LoT category (endocrine and targeted therapy categories combined) showing proportion of patients dying following treatment at each LoT. Treatment categories beyond a third treatment are not shown

The sequence of chemotherapy and endocrine (with/without targeted) therapy received by patients from 1st to 3rd LoT was diverse (Fig. 2a). Of the 185 patients who received 1st LoT, 171 (92.4%) received endocrine (with/without targeted) therapy and 85 (45.9%) received chemotherapy at some time during the study period. When given, 2nd LoT was more often endocrine (with/without targeted) therapy (*n* = 96, 77.4%) than chemotherapy (*n* = 28, 22.6%) (Fig. 2a). When given, 3rd LoT was again more often endocrine (with/without targeted) therapy (*n* = 49, 65.3%) than chemotherapy (*n* = 26, 34.7%).

There was substantial diversity in the sequence of SACT classes and regimens used (Additional file 2). One line of endocrine therapy with a single agent AI, was the treatment sequence used most frequently (27.6%, *n* = 51 patients). The next most common were three successive lines (*n* = 19, 10.3%) and two successive lines (*n* = 17, 9.2%) of endocrine therapy, each without targeted therapy. The fourth most common treatment sequence was the first to include chemotherapy, (3rd LoT capecitabine following endocrine therapy; *n* = 8, 4.3%).

Patients receiving 1st LoT chemotherapy were more likely than those receiving endocrine (with/without targeted) therapy to receive subsequent chemotherapy (26.1 and 11.5%, respectively; *p =* 0.02: Fig. 2b). Regardless of 1st LoT modality, all patients receiving 2nd LoT chemotherapy either subsequently received further treatment or died. In contrast, 18 patients (18.8%) receiving 2nd LoT endocrine (with/without targeted) therapy remained alive with no further change of treatment until study end.

### Overall survival

**Fig. 3:**
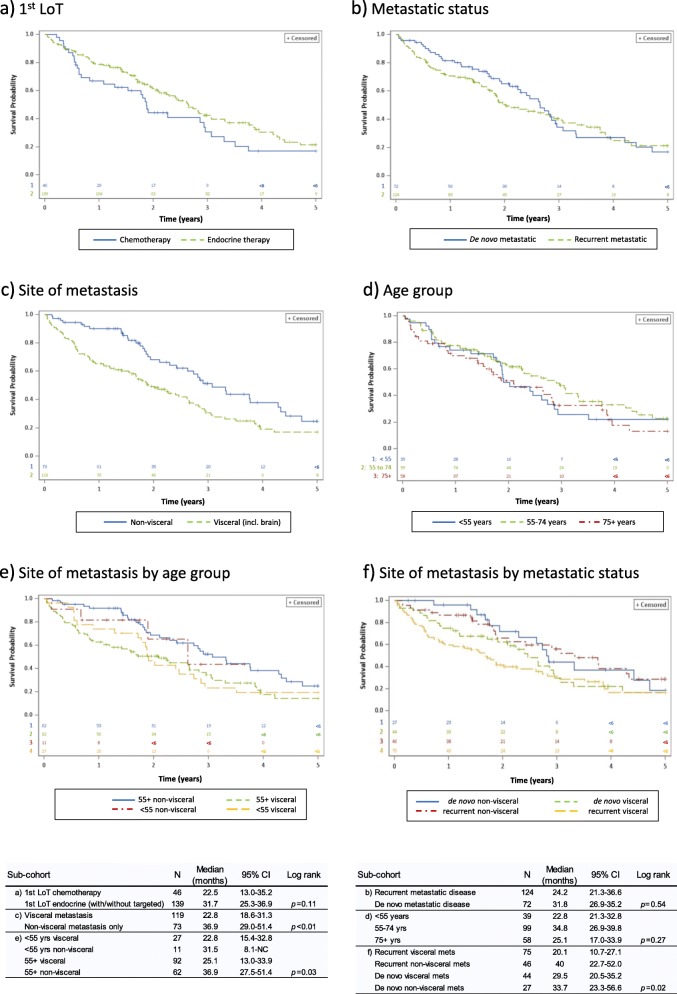
Five-year OS all causes by (**a**) modality of 1st LoT (*n* = 181), **b** de novo or recurrent metastatic status (*n* = 196), **c** site of metastasis (*n* = 192), **d** age group (*n* = 196), **e** site of metastasis by age group (*n* = 192) and **f** site of metastasis by metastatic status (*n* = 192)

Median OS for the study cohort (*n* = 192) following diagnosis of metastatic disease was 29.5 months (95% CI: 23.3–34.4). For patients receiving 1st LoT chemotherapy, median OS was 22.5 months compared to 31.7 months for those receiving endocrine (with/without targeted) therapy (*p* = 0.11) (Fig. 3). Median OS was 31.8 months in those with de novo MBC compared to 24.2 months in those with recurrent MBC (*p* = 0.54); the apparent early survival benefit in patients with de novo MBC disappeared after 36 months (Fig. 3). By contrast, OS was significantly better for patients with exclusively non-visceral metastasis than those with visceral metastasis (median 36.9 months and 22.8 months, respectively; *p* < 0.01) and this benefit appeared to be regardless of whether patients had de novo or recurrent MBC (*p =* 0.02) and age group (*p =* 0.03) (Fig. 3). OS was particularly poor for patients with recurrent MBC who had visceral metastasis (20.1 months).

Given the observed associations of young age, visceral metastasis, presentation with recurrent disease and 1st LoT chemotherapy, a Cox PH analysis was undertaken to further investigate overall survival. Site of metastasis and modality of 1st LoT were highly correlated (*p* < 0.01) and therefore prone to collinearity in the regression model; modality of 1st LoT was subsequently dropped from the final model. Presentation with recurrent disease was not a significant explanatory variable for survival and was also dropped, leaving site of metastasis and age. There were 181 patients available for analysis and 72 patients (39.8%) were censored. Age (*p* = 0.04) and site of metastasis (*p* < 0.01) were both significant determinants of OS in the combined model; when adjusted for age, the HR for death for patients with visceral metastasis was 1.91 (95% CI: 1.27–2.89) relative to patients with non-visceral metastasis only.

## Discussion

This real-world study of patients with HR+/HER2- MBC between 2012 and early 2018 who had not participated in a clinical trial is relevant in understanding the potential future changes in clinical practice at a time when treatment options are developing rapidly (especially the introduction of CDK 4/6 inhibitors). In this single-centre population, almost 70% of patients newly diagnosed with advanced or metastatic MBC had HR+/HER2-. Approximately two thirds of patients had recurrent MBC; the remaining third had de novo MBC and therefore, by definition, had not received previous SACT.

As expected, and in line with guidelines, almost all patients (98%) received endocrine therapy at some point and for three quarters of them, this was the 1st line of SACT. These patients most frequently received an AI, commonly letrozole (perhaps reflecting the local use of anastrazole in the (neo)adjuvant setting). The importance of endocrine therapy in the treatment of HR+/HER2- MBC is reinforced by the finding that the three most frequently used sequences of SACT comprise endocrine therapies alone. The lower proportion of endocrine-treated patients receiving further SACT (compared with chemotherapy-treated) most likely reflects the fact that those starting endocrine therapy will continue with it to disease progression, whereas those treated with chemotherapy may well switch to endocrine maintenance prior to progression.

Approximately half (47.5%) of patients received cytotoxic chemotherapy, and a quarter received chemotherapy as 1st LoT. The proportion of patients receiving 1st LoT chemotherapy decreased over the study period from greater than 30% to less than 20%; although not statistically significant, this trend is of interest and worthy of further study. In a real-world study from the Netherlands, a quarter of patients with HR+ MBC between 2007 and 2009 received 1st LoT chemotherapy [19]; the figure was higher in similar studies from Italy (42%) [20], US (40%) [21] and Japan (43%) [22], suggesting differences in, and evolution of, practice over time between countries, despite the consistency of guidelines.

The use of 1st LoT chemotherapy does not contravene guidelines but is likely to indicate a patient group with rapidly progressive disease that remained fit enough to receive it. Indeed, chemotherapy was used as 1st LoT especially in younger women with visceral disease; a group potentially more likely to tolerate and benefit from such treatment. The somewhat greater use of 1st LoT chemotherapy in patients with recurrent MBC compared with de novo MBC may reflect the higher incidence of visceral disease in the former group. An MFI less than 5 years (usually indicating relapse whilst on (neo)adjuvant endocrine therapy for patients with recurrent MBC) was also associated with 1st LoT chemotherapy. Patients with short MFI in a study from the Netherlands had particularly poor survival [23], but analyses of treatment choices were not reported.

Perhaps more surprising, although single agent capecitabine and weekly paclitaxel were the most widely used cytotoxics, almost half 1st LoT chemotherapy was given in combination, whereas guidelines generally recommend sequential single agent chemotherapy. Patient numbers were small, but carboplatin/paclitaxel was given only to those with recurrent disease and prior chemotherapy treatment and EC chemotherapy was given only to patients with visceral disease. These associations suggest that oncologists are “personalising” their use of chemotherapy as first line SACT. The heterogeneous treatment patterns resulting from use of both endocrine therapy (with/without targeted therapy) and chemotherapy across multiple LoT further reinforces the conclusion that treatment of HR+/HER2- MBC is very much personalised in the real-world setting. This approach can be expected to increase with the advent of new treatment options in the form of CDK4/6 inhibitors [24] and the anticipated availability of PI3K inhibitors [25].

After each LoT a significant proportion of patients in this study did not receive further treatment: some remained on their current treatment, but others died or will have been unwilling or unable to receive SACT. This attrition through subsequent lines of treatment emphasizes the importance of utilizing the most effective and best tolerated SACT sooner rather than later. This may become more important with the introduction of CDK4/6 inhibitors, where subsequent delays in the requirement for chemotherapy treatment combined with lesser impact on OS compared to PFS, may limit the time for subsequent lines of treatment, be they endocrine (with/without chemotherapy) or chemotherapy [10, 11]. As yet, there is a paucity of trial data on the efficacy of SACT following 1st LoT endocrine therapy combined with a CDK4/6 inhibitor. Preliminary real-world experience suggests that chemotherapy remains effective and tolerable following a CDK4/6 inhibitor but there is a need for more data [26].

Median OS in this real-world study cohort was approximately 30 months following the diagnosis of MBC and worse for those receiving 1st LoT chemotherapy (23 months) than for those receiving endocrine (with or without targeted) therapy (32 months), although this difference did not reach statistical significance in our study. In the study from the Netherlands, those initially receiving chemotherapy also had worse OS (16 months compared with 37 for those receiving endocrine) and this was significant [19] but an Italian study showed the reverse (38 months OS following 1st LoT chemotherapy compared with 33 months following endocrine therapy) [27]. Differences between countries may reflect patient mix and patterns of follow up after diagnosis of early BC.

Shorter OS for patients receiving chemotherapy as 1st LoT most likely reflects their more aggressive underlying disease as noted above. Indeed, presence of visceral metastasis was highly correlated with 1st LoT chemotherapy in our study and associated with worse OS (23 months for patients with visceral metastasis compared with 37 months for those with non-visceral metastasis). In a Cox regression analysis, it was the sites of metastasis that proved to be the dominant factor influencing OS, though we acknowledge that the total number of observations is small and this model may have low power.

The apparently worse outcomes seen with chemotherapy emphasize the limitations of chemotherapy in this patient group. Indeed, in our study 30% of patients treated with first line chemotherapy did not subsequently receive an endocrine agent. Moreover, it suggests that there may be significant benefits if endocrine therapy was used more frequently and earlier for patients with more aggressive HR+/HER2- MBC. The efficacy and tolerability of 1st LoT SACT with endocrine therapy and the emerging use of CDK4/6 inhibitors suggests that the trend towards falling use of chemotherapy observed in this setting may indeed continue.

This study has both strengths and weaknesses. Strengths include it being a single centre study of patients treated by a single team of non-surgical oncologists. The period over which patients were treated is also relevant when gauging the treatment landscape prior to the introduction of CDK4/6 inhibitors. The main limitation of the study is that, coming from a single centre, the size of our patient cohort was limited; this enabled us, however, to gather data both from the EPR directly and by inspection. Including patients diagnosed with MBC before 2012 would have increased patient numbers but at the price of the cohort being less contemporary and potentially less relevant. Including patients treated within a clinical trial (prior to or during the study period) would also have increased the cohort, but this was not compatible with original study aims to describe routine real-world clinical practice. The US real world study was much larger, but was based on an insurance claim database and collected limited clinical data [21]. We collected more data, but the study was not designed to characterise aspects of treatment such as whether endocrine treatment following chemotherapy was being used as “maintenance” treatment or because of disease progression.

Real-world studies complement well designed clinical trials and allow for cost-effective analysis of large and representative patient populations, many of whom would be excluded from clinical trials. They provide an important perspective on clinical practice and patient outcomes in ordinary healthcare settings. Rapid developments in EPRs and the ability to link clinical informatics in primary and secondary care, raise the prospect of integrating databases such that information on other explanatory variables such as co-morbidities and social factors becomes available at scale. There is also the potential to look at how real-world outcomes evolve with the introduction of new treatments and to gauge their wider societal impact. For example, the ongoing Epidemiological Strategy and Medical Economics (ESME) research programme is centralising real-life data on more than 16,000 patients with BC from a single French Comprehensive Cancer Centre for research purposes [28].

In conclusion, this study demonstrates the heterogeneous patterns of SACT treatment of patients with HR+/HER2- MBC exclusive of clinical trial involvement. Looking ahead, there is opportunity for increased use of combination endocrine therapy as 1st LoT, delaying the introduction of chemotherapy and perhaps limiting the number of lines of subsequent treatment. This further emphasizes the need to optimise use of the most effective and best tolerated SACT earlier in the natural history of MBC. We plan to repeat this work in a future cohort of patients treated after CDK4/6 inhibitors became available through the NHS.

Supplementary information is available at the BMC Cancer website.

## Conclusions

In real-world clinical practice, diverse endocrine therapies predominate as 1st LoT for patients with HR+/HER2- MBC. Use of chemotherapy as 1st LoT is associated with more aggressive disease in younger patients. In a context of personalised treatment, use of effective and tolerable therapies to treat MBC early is important.

## Supplementary information

**Additional file 1.** STROBE flow chart of study inclusion.

**Additional file 2.** The three most common sequence of regimens received by patients (*n* = 185) in each major treatment sequence category, showing the total number of patients in each sequence category and the proportion of all treated patients (small number masking in operation).

## Data Availability

Data available on request from the authors.

## Abbreviations

AJCC: American Joint Committee on Cancer
CDK: Cyclin-dependent kinase
EPR: Electronic patient records
HER2-: Human epidermal growth factor 2 negative
HR: Hazards ratio
HR+: Hormone receptor positive
ICD: International Classification of Disease
LCC: Leeds Cancer Centre
LoT: Line of therapy
LTHT: Leeds Teaching Hospitals Trust
MBC: Metastatic breast cancer
MFI: Metastasis free interval
OS: Overall survival
PFS: Progression free survival
PH: Proportional hazards
PPM: Patient pathway manager
REAL: Real-world Evidence Alliance Leeds
SACT: Systemic anticancer treatment
