# Supplementary figures and images for: Systemic treatment of hormone receptor positive, human epidermal growth factor 2 negative metastatic breast cancer: retrospective analysis from Leeds Cancer Centre
Source: BMC Cancer. 2020 Jan 21;20:53. doi: 10.1186/s12885-020-6527-y (PMC6975018; doi:10.1186/s12885-020-6527-y)

## Supplementary 1 STROBE flow chart for study inclusion

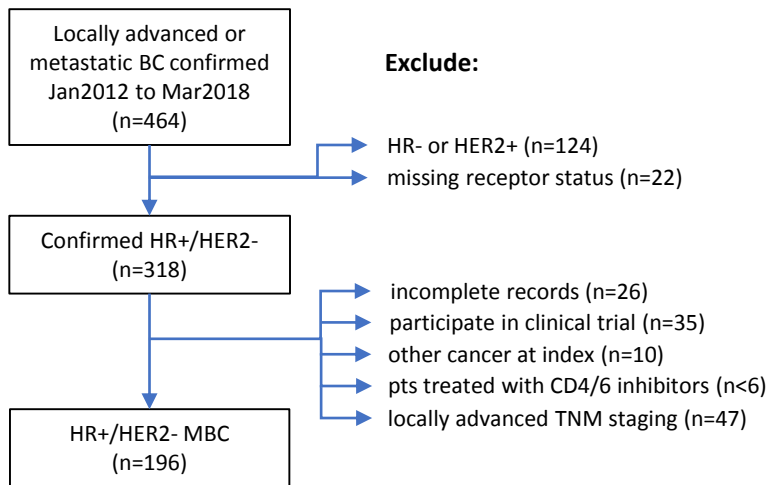

Supplement: Supplementary file 1 — Additional file 1. STROBE flow chart of study inclusion. [file 12885_2020_6527_MOESM1_ESM.pdf]
