# Supplementary material for: Systemic treatment of hormone receptor positive, human epidermal growth factor 2 negative metastatic breast cancer: retrospective analysis from Leeds Cancer Centre
Source: BMC Cancer. 2020 Jan 21;20:53. doi: 10.1186/s12885-020-6527-y (PMC6975018; doi:10.1186/s12885-020-6527-y)
Supplement: Supplementary file 2 — Additional file 2. The three most common sequence of regimens received by patients (n = 185) in each major treatment sequence category, showing the total number of patients in each sequence category and the proportion of all treated patients (small number masking in operation). [file 12885_2020_6527_MOESM2_ESM.pdf]

Supplementary 2 The three most common sequence of regimens received by patients (n=185) in each major treatment sequence category, showing the total number of patients in each sequence category and the proportion of all treated patients (small number masking in operation).

Sequence	Agents(s)
Endo	Letrozole
(n=51, 27.6%)	Anastrozole
	Exemestane
Endo - Endo - Endo	Letrozole - Exemestane – Tamoxifen
(n=19, 10.3%)	Letrozole - Fulvestrant - Tamoxifen
	Exemestane - Tamoxifen - Letrozole
Endo - Endo	Letrozole – Exemestane
(n=17, 9.2%)	Letrozole – Tamoxifen
	Anastrozole – Exemestane
Endo - Endo - Chemo	Exemestane - Fulvestrant - Capecitabine
(n=8, 4.3%)	Letrozole - Exemestane - Capecitabine
	Anastrozole - Fulvestrant - Capecitabine
Chemo	Paclitaxel
(n=7, 3.8%)	EC
Endo - Endo/Targ - Endo	Letrozole - Evero/Exem – Tamoxifen
(n=7, 3.8%)	Letrozole - Evero/Exem - Fulvestrant
	Anastrozole - Evero/Exem - Fulvestrant
Chemo - Chemo	Carboplatin – Capecitabine
(n=6, 3.2%)	Paclitaxel – Carboplatin
	EC – Paclitaxel
Endo - Chemo - Chemo	Exemestane - Capecitabine - Paclitaxel
(n<6)	Letrozole - Paclitaxel - Capecitabine
	Anastrozole - Capecitabine - Paclitaxel
Endo - Endo/Targ	Anastrozole - Evero/Exem
(n<6)	Letrozole - Evero/Exem
	Tamoxifen - Evero/Exem
Chemo - Endo - Endo	EC - Letrozole - Exemestane
(n<6)	Capecitabine - Tamoxifen - Fulvestrant
	Paclitaxel - Letrozole - Anastrozole
Chemo - Endo - Chemo	EC - Letrozole - Paclitaxel
(n<6)	Capecitabine - Tamoxifen - Paclitaxel
	Carboplatin - Letrozole - Paclitaxel
Chemo - Chemo - Endo	EC- Docetaxel - Tamoxifen
(n<6)	Carboplatin - Paclitaxel - Anastrozole
	Docetaxel - Capecitabine - Fulvestrant
